# Zinc and Zinc Transporters in Macrophages and Their Roles in Efferocytosis in COPD

**DOI:** 10.1371/journal.pone.0110056

**Published:** 2014-10-28

**Authors:** Rhys Hamon, Claire C. Homan, Hai B. Tran, Violet R. Mukaro, Susan E. Lester, Eugene Roscioli, Mariea D. Bosco, Chiara M. Murgia, Margaret Leigh Ackland, Hubertus P. Jersmann, Carol Lang, Peter D. Zalewski, Sandra J. Hodge

**Affiliations:** 1 Discipline of Medicine, University of Adelaide, The Queen Elizabeth Hospital, Woodville, South Australia, Australia; 2 Department of Thoracic Medicine, Royal Adelaide Hospital, Lung Research Laboratory, Hanson Institute, Adelaide, South Australia, Australia; 3 Rheumatology Unit, The Queen Elizabeth Hospital, Woodville, South Australia, Australia; 4 Discipline of Surgery, University of Adelaide, The Queen Elizabeth Hospital, Woodville, South Australia, Australia; 5 CRA-NUT, Roma, Italy; 6 Centre for Cellular and Molecular Biology, School of Life and Environmental Sciences, Deakin University, Burwood, Melbourne, Victoria, Australia; The Florey Institute of Neuroscience and Mental Health, Australia

## Abstract

Our previous studies have shown that nutritional zinc restriction exacerbates airway inflammation accompanied by an increase in caspase-3 activation and an accumulation of apoptotic epithelial cells in the bronchioles of the mice. Normally, apoptotic cells are rapidly cleared by macrophage efferocytosis, limiting any secondary necrosis and inflammation. We therefore hypothesized that zinc deficiency is not only pro-apoptotic but also impairs macrophage efferocytosis. Impaired efferocytic clearance of apoptotic epithelial cells by alveolar macrophages occurs in chronic obstructive pulmonary disease (COPD), cigarette-smoking and other lung inflammatory diseases. We now show that zinc is a factor in impaired macrophage efferocytosis in COPD. Concentrations of zinc were significantly reduced in the supernatant of bronchoalveolar lavage fluid of patients with COPD who were current smokers, compared to healthy controls, smokers or COPD patients not actively smoking. Lavage zinc was positively correlated with AM efferocytosis and there was decreased efferocytosis in macrophages depleted of Zn *in vitro* by treatment with the membrane-permeable zinc chelator TPEN. Organ and cell Zn homeostasis are mediated by two families of membrane ZIP and ZnT proteins. Macrophages of mice null for ZIP1 had significantly lower intracellular zinc and efferocytosis capability, suggesting ZIP1 may play an important role. We investigated further using the human THP-1 derived macrophage cell line, with and without zinc chelation by TPEN to mimic zinc deficiency. There was no change in *ZIP1* mRNA levels by TPEN but a significant 3-fold increase in expression of another influx transporter *ZIP2,* consistent with a role for ZIP2 in maintaining macrophage Zn levels. Both ZIP1 and ZIP2 proteins were localized to the plasma membrane and cytoplasm in normal human lung alveolar macrophages. We propose that zinc homeostasis in macrophages involves the coordinated action of ZIP1 and ZIP2 transporters responding differently to zinc deficiency signals and that these play important roles in macrophage efferocytosis.

## Introduction

The phagocytic activity of macrophages is important not only for immune response to micro-organisms but also for removal of apoptotic cells in tissues in a process known as efferocytosis (Latin, *efferre*, “to carry to the grave”) [1]. Impairment of efferocytosis can lead to accumulation of apoptotic cells and their secondary necrosis, releasing factors that promote an inflammatory response. Efferocytosis is impaired by a number of agents including cigarette smoke and is an important pathogenic mechanism in chronic inflammatory diseases of the lung such as chronic obstructive pulmonary disease (COPD) [2]–[7]. There is interest, therefore, in factors which regulate efferocytosis.

One such factor is zinc (Zn), the second most abundant metal in the body and important for many physiological and pathological processes, especially in the immune system. Increased prevalence of obstructive lung disorders [8] and lung (but not breast or prostate) cancer [9] are associated with low dietary Zn intake and thought to be due, at least in part, to protective effects of Zn against cadmium, a metal ion with similar chemistry to Zn but which is toxic and accumulates in AM of smokers [10]. Amongst the actions of Zn ions on the immune system are its effects on phagocytic cells including monocytes, macrophages and dendritic cells [11]–[13]. Phagocytosis is impaired in Zn deficient mice and restored by Zn supplements [14], [15]. Studies from Guidot and colleagues [16], [17] have suggested links between lung injury, impaired phagocytosis and Zn deficiency. In rats, stresses to the lung caused by alcohol [17] or virus [16], [18] resulted in significant decreases in GM-CSF receptor expression and phagocytosis of bacteria by AM. These were accompanied by a significant decline in lung Zn concentrations, as measured by Zn in BAL fluid supernatant and AM. A causative role for Zn deficiency in macrophage impairment is supported by decrease in AM phagocytosis of bacteria by chelation of intracellular Zn *in vitro* induced by the membrane-permeable Zn chelator N,N,N',N'-tetrakis-(2-pyridyl-methyl) ethylenediamine (TPEN). In addition, alcohol-fed rats had a 5-fold decrease in capacity to clear inoculated *Klebsiella pneumonia* from their lungs and this, as well as increased oxidative stress in the lung, could be prevented by dietary zinc supplementation [19]. These findings were extended to human chronic alcoholic subjects who had significantly decreased AM Zn levels, bacterial phagocytosis and expression of GM-CSF receptor; treating AM with Zn *in vitro* improved their phagocytic function [20]. Collectively, these studies showed that Zn is required to maintain AM bacterial phagocytosis, and that pulmonary Zn deficiency could be one of the mechanisms by which chronic HIV-1 infection and alcohol abuse impair AM immune function and predispose to pneumonia and other lung infections.

Little is known about Zn in macrophage efferocytosis of apoptotic cells and its effects in chronic lung diseases including COPD. It is also not known how macrophages regulate their levels of Zn including their responses to Zn deficiency. Critical to the normal cellular and tissue homeostasis of Zn is the role of specific membrane Zn transporter proteins. Based on their sequence homology and structural properties, Zn transporters have been assigned to two families: SLC39A (also known as ZIPs) and SLC30A (also known as ZnTs) [21]. ZIPs usually have 8 trans-membrane domains and either mediate uptake of Zn across the plasma membrane (e.g. ZIP4) or regulate release of Zn into the cytosol from organelles such as the endoplasmic reticulum and Golgi (e.g. ZIP7). They are particularly important in restoring normal cytosolic levels of Zn in Zn-deprived cells. ZnTs generally have 6 trans-membrane domains and have opposing actions, lowering cytosolic Zn by facilitating efflux from cells (e.g. ZnT1) or uptake of the metal ions from cytosol into intracellular organelles (e.g. ZnT4). A range of Zn transporters are expressed in the lung [22], including special roles for three influx membrane ZIP transporters. ZIP1 is lost, along with Zn, in the lungs of rats with acute lung injury [17]. ZIP8 is increased at mRNA and protein levels in the lungs of chronic smokers and may play a pivotal role in the ensuing damage since, on the one hand, it transports Zn which protects airway epithelium and is anti-inflammatory in the lungs [23], [24] while it also transports cadmium (Cd) a toxic metal in inhaled cigarette smoke that accumulates in the lungs [25]. ZIP14 is a major influx transporter for Zn in pulmonary endothelium [26].

If efferocytosis is Zn-dependent, this might explain our earlier observations of a marked accumulation of apoptotic epithelial cells in the lungs of mice with combined airway inflammation and nutritional Zn deprivation as well as reduced efferocytosis in the airway and lungs of patients with COPD and smoke exposed mice [2], [4], [5], [27]. What is required now is a systematic characterization of Zn homeostasis in macrophages and a study of its role in efferocytosis. We firstly assessed Zn levels in BAL of human COPD subjects and healthy subjects, with and without a recent history of smoking, and correlated with AM efferocytosis activity. Next we assessed the effects of Zn depletion on efferocytosis in AM. To investigate the effect of Zn depletion on gene expression of ZIP transporters, we used human THP-1 macrophages [28]. Confirmation of protein expression and subcellular localization of ZIP transporter proteins was determined by immunofluorescence in THP-1 cells and normal human lung sections. Further investigation of the role in efferocytosis of one of these transporters ZIP1 was carried out using ZIP1 null mice [29].

## Materials and Methods

### Subject populations and collection of samples

Human ethics approval was granted by the institutional review board for human studies at the Royal Adelaide Hospital (RAH), which approved the protocols; all procedures were undertaken after written fully informed consent from the subjects. BAL was collected from controls (normal lung function and no history of lung disease, cancer or allergy) and COPD subjects who were further sub-classified according to smoking history [2]–[4], [6]. AM were purified by adhesion to plastic as we have reported [4]. Primary lung tissue was obtained from a separate group of patients undergoing curative-intent lobectomy as previously reported [30], [31]. All COPD subjects had moderate severity disease (GOLD stage 2) and there were no significant associations between disease severity and Zn levels among these subjects.

### Materials

Zn sulphate (cell culture grade), ovalbumin, TPEN, PMA and DAPI and ß-mercaptoethanol were from Sigma-Aldrich (Sydney, NSW, Australia). Other reagents were obtained as described below or in the text.

### Zn measurement in human bronchoalveolar fluid (BAL)

BAL were centrifuged and supernatants frozen at –80°C until assayed for Zn. BAL Zn was assayed by a Zinquin-based fluorometric technique [32] as follows. Briefly, 100 µL of BAL samples (or stock Zn sulphate solutions) were added in triplicates to wells of 96-well black plates (BD Biosciences, North Ryde, NSW, Australia). To these were added 100 µL of Zinquin Assay buffer (Chelex treated, Zn-free 1X Hank’s balanced salt solution with 0.3 mg/ml ovalbumin and 10 µM Zinquin ester. After 40 min incubation at room temp in the dark, fluorescence was read using excitation filter (355 nm) and emission filter (510–10 nm) in a FLUOstar Optima fluorescence plate reader (BMG Labtech, Mornington, Victoria, Australia) and converted to Zn concentration from a standard curve.

### THP-1 cell culture

A differentiated THP-1 human monocytic cell line (ATCC, Rockville, MD) was applied as these cells behave very much like native monocyte-derived macrophages and have been used extensively to study mechanisms involved in human macrophage differentiation [28]. Cells were cultured in RPMI 1640 media supplemented with 2 mM GlutaMax™ 10% fetal calf serum (FCS) and 1% Penicillin:Streptomycin (all from Life Technologies, Carlsbad, CA) and 0.05 mM ß-mercaptoethanol. THP-1 cells (1×10^6^) were differentiated into macrophages in 6-well plates in 2 mL of RPMI 1640 medium containing 0.1 µM of PMA over 48 hr at 37°C. Differentiation was assessed by adherence to plastic. and determination of plasma membrane CD11B expression using flow cytometry as described [33]. To deplete intracellular Zn, aliquots of cells were treated for 4 h with 16 µM of TPEN. Since TPEN is a known apoptotic inducer of cells [34], a loss in cell viability in TPEN-treated cells could potentially confuse interpretation of some of the findings. The effect of TPEN treatment on apoptosis of THP-1 macrophages was assessed using flow cytometry and 7AAD staining. The percentage apoptosis of cells both pre-and post- treatment was less than 1%. Therefore, under the conditions (concentration and exposure time) used in this study, there was no effect of TPEN on apoptosis.

### Flow cytometric measurement of labile Zn using FluoZin-3

AM, THP-1 cells or PMA treated THP-1 cells (scraped from culture dish) were loaded with FluoZin-3 AM ester (1 µM, Life Technologies) in culture medium at 37°C for 30 min, washed with 1 mL of FACS buffer (1X PBS, 0.5% BSA, 2 mM EDTA) and resuspended in 100 µL of FACS buffer. Cells were loaded with FluoZin-3 and incubated with anti-CD11b conjugated with *phycoerythrin* (PE) for 30 min on ice in the dark. Fluorescence was recorded using a FACS Canto II (BD). Cells were gated relative to an unstained control and the percentage of cells that stained positive was determined. The following controls were used to determine compensation between FITC and PE channels: unstained cells to determine appropriate FSC and SSC PMT settings, and cells loaded with FluoZin-3 AM ester alone and cells stained with CD11b PE alone to determine compensation between both channels. BAL Zn levels were not considered an issue as macrophages were washed twice before the assay and cells further washed with FACS buffer before staining with CD11b PE.

### qRT-PCR

Cells were immediately placed in RLT lysis buffer (Qiagen, Chadstone Centre, Victoria, Australia). Total RNA was isolated as per kit instructions (RNeasy mini kit, Qiagen). cDNA was prepared from 2 µg of total RNA using a high capacity cDNA reverse transcription kit (Applied Biosystems, Mulgrave, Victoria, Australia). Quantitative PCR was performed using 33 ng of cDNA as template in 10 µL reactions using pre-made TaqMan primer/probes (Applied Biosystems) with TaqMan gene expression master mix and run on an Applied Biosystems ViiA7 Real Time PCR system. Hypoxanthine guanine phosphoribosyl transferase (HPRT) and 18S rRNA were used as endogenous controls. Data was analysed by the delta-delta Ct method, and expressed as fold change (with 95% confidence intervals).

### Immunofluorescence antibodies, reagents and fluorescence microscopy

Immunofluorescence of Zn transporters in THP-1 cells or formalin-fixed lung biopsies was performed as described for inflammasome proteins [35]. Human ZIP1 polyclonal antibody was raised in sheep [36]. Primary antibodies, other than ZIP1, were from commercial sources including rabbit polyclonal antibodies to human ZIP2 (Abcam74707, Sapphire Bioscience, Waterloo, NSW; Australia). In some experiments, sections were co-stained for CD68 (PG-M1, Dako Australia Pty. Ltd, Nth Sydney, NSW, Australia) or DAPI (200 ng/mL). F(ab’)2 fragment secondary antibodies were from Jackson ImmunoResearch (Abacus ALS, Brisbane, QLD, Australia). Cytospins were fixed with 2.5% formalin and permeabilized with 0.1% SDS. Images were captured on a Zeiss Apoptome microscope (Carl Zeiss GmbH, Goettingen, Germany).

### Efferocytosis assay

Efferocytosis of apoptotic epithelial cells by macrophages was performed as we have previously described using apoptotic human or mouse lung epithelial cells at a ratio of 1:10 macrophages:apoptotic cells as phagocytic targets [5], [6]. The efferocytic index was determined as the percentage of macrophages ingesting apoptotic cells.

### Animal Studies

The Animal Care and Ethics Committees of the IMVS and the University of Adelaide approved animal procedures (Permits M-37-08 and M-67-09) and procedures conformed to ARRIVE guidelines, NIH guidelines (Guide for the Care and Use of Laboratory Animals. NIH publication No. 86–23) and to National Health and Medical Research Guidelines to promote the wellbeing of animals used for scientific purposes: The assessment and alleviation of pain and distress in research animals (2008). In our experiments, all efforts were made to minimize suffering of the mice. Prior to obtaining peritoneal macrophages, mice were anaesthetized with isofluorane and humanely killed by cervical dislocation. ZIP1^−/−^ mice were provided by Jim Geiser and Glen Andrews (Univ of Kansas Medical Center, Kansas) [29] and back-crossed to a C57BL/6s background. Matings of heterozygotes provided the ZIP1^−/−^ null mice and ZIP1^+/+^ wild-type controls. Mice were maintained in the the Institute of Medical and Veterinary Science (IMVS, Gilles Plains, Australia) animal care SPF facility (22°C with a 12 h light-dark cycle). Access to water and a non-purified diet (Milling Industries, Adelaide, Australia) were provided ad libitum.

### Isolation of Mouse Peritoneal Macrophages and Phagocytosis Assay

For good yield of macrophages we obtained peritoneal macrophages. Female mice (average size 22 g), randomly selected from null or wild-type controls, were injected *ip* with 1 mL of **4**% Brewer’s Thioglycollate broth (Sigma-Aldrich, Nth Ryde, NSW). After 2 days, mice were sacrificed (see above) prior to obtaining peritoneal macrophages. Cells were centrifuged at 800 RPM and seeded (3×10^5^ cells) into wells of 24-well plates in RPMI+GlutaMax™ with 10% foetal bovine serum and 1% Penicillin:Streptomycin. Plates were incubated for 2 hrs to allow macrophages to adhere to the plate. Non-adherent cells were removed and adherent macrophages resuspended in RPMI+Glutamax™ medium. Phagocytosis of apoptotic murine epithelial cells by mouse peritoneal macrophages was performed as previously described [5], [6].

### Statistical analysis

Data was analysed using SPSS software and Mann-Whitney or 1-way ANOVA with post hoc for nonparametric analyses. Correlations were performed using Spearman’s rank test. Analyses were performed using SPSS software. P values<0.05 were considered significant.

## Results

### 1. Zn concentrations in human BAL

**Figure 1:**
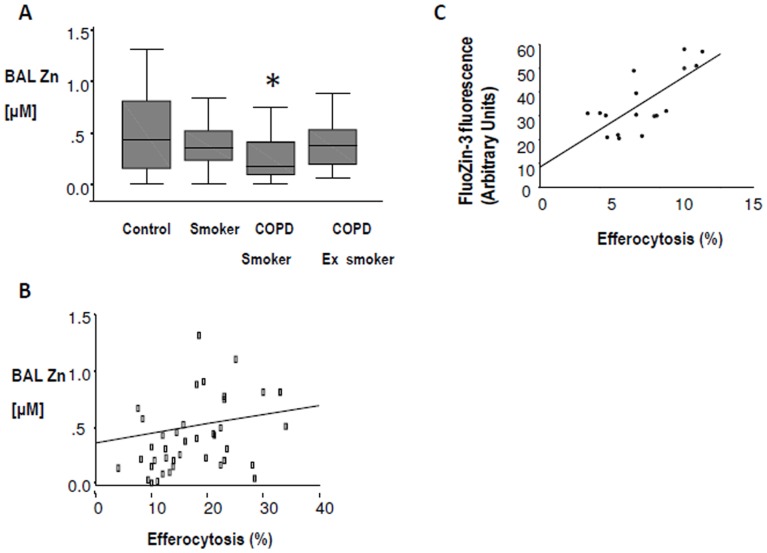
Relationship between efferocytosis and BAL and macrophage Zn content. **A:** Zn levels in BAL from COPD and smokers. Zn levels were determined in BAL from 20 controls, 17 healthy smokers, 20 current and 19 ex-smoker COPD subjects. Box plots present median ±25th and 75th percentiles (solid box) with the 10th and 90th percentiles shown by whiskers outside the box. Asterix indicates significantly (p<0.05) lower expression vs. controls. **B:** Correlation between BAL Zn levels and % efferocytosis. A significant correlation between BAL Zn levels and % efferocytosis of apoptotic bronchial epithelial cells was also found (correlation coefficient 0.345, p = 0.025, Spearman’s rho). **C:** Correlation between intracellular Zn levels and % efferocytosis. Figure shows a strong positive correlation between intracellular labile Zn levels (measured by FluoZin-3 fluorescence) and % efferocytosis in patients’ AM.

Zn levels were determined in BAL from 20 controls, 17 healthy smokers, 20 current and 19 ex-smoker COPD subjects. Mean BAL Zn concentration was significantly decreased in COPD patients who were current smokers, compared to controls p<0.05 (Mann Whitney, Fig. 1A). Mean BAL Zn concentrations were lower in healthy smokers and ex-smoker COPD subjects compared to controls, but p values did not reach significance. There was no significant correlation between age and BAL Zn concentration amongst those with COPD (Pearson Correlation, p = −0.125).

### 2. Zn and AM efferocytosis

Correlations were performed on a cohort of 38 subjects for which both efferocytosis and Zn data were available (Fig. 1B). Further correlations were performed on a cohort of 17 subjects for which both efferocytosis and intracellular labile Zn levels in patients’ AM were available (Fig. 1C). The groups contained normal, smoker, COPD smoker and COPD ex-smokers at similar proportions to those in Figure 1A. A significant correlation between BAL Zn levels and % efferocytosis of apoptotic human 16HBE bronchial epithelial cells was found (n = 38, correlation coefficient 0.345, p = 0.025, Spearman’s rho) (Fig. 1B). There was strong correlation between intracellular labile Zn levels in patients’ AM (measured by FluoZin-3 fluorescence) and % efferocytosis (correlation coefficient 0.722, p = 0.001, Fig. 1C).

### 3. Changes in Zn transporter expression during TPEN-induced Zn depletion of THP-1 cells

**Figure 2:**
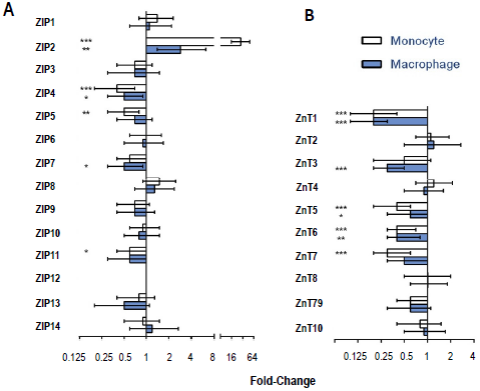
Zn transporter mRNA expression in THP-1 monocytes and macrophages in response to intracellular Zn depletion. THP-1 monocytes and PMA differentiated THP-1 macrophages were treated with 16 µM TPEN for 4 h. RNA was isolated and target genes were detected using Taqman probes, with HPRT-1 and 18S RNA used as endogenous controls for 3 separate experiments (n = 3). Data is presented as mean fold change (±95% CI) compared to control (untreated) cells. * p: <0.05, ** p: <0.01, *** p: <0.001.

To mimic Zn deficiency, THP-1 monocytes or macrophages were treated for 4 h with the membrane permeable Zn chelator TPEN. The effects of TPEN on mRNA expression for the Zn transporters and other Zn-related proteins are shown in Fig. 2 as mean values with 95% confidence intervals.

#### a) ZIP family of Zn transporters

*ZIP2* mRNA significantly increased in TPEN-treated THP-1 monocytes (28.3-fold, p<0.001) and THP-1 macrophages (2.9-fold (p<0.005). TPEN significantly decreased mRNA levels of *ZIP4* in both monocytes (0.4-fold, p<0.001) and macrophages (0.5-fold, p<0.05). There were similar decreases in *ZIP7* but these only reached significance in macrophages (0.5-fold, p<0.05); in monocytes, p value was 0.065. *ZIP11* was significantly decreased in monocytes (0.6-fold, p<0.05) but not in macrophages. There were no effects of Zn depletion by TPEN on expression of the other nine ZIP transporters (*ZIP1,3,5,6,8,9,10,13,14*). *ZIP12* was not expressed in either cell type.

#### b) ZnT family of Zn transporters

TPEN significantly decreased mRNA expression of *ZnT1* (0.2-fold, p<0.001) and *ZnT6* (0.4-fold, p<0.002) in both monocytes and macrophages. *ZnT5* mRNA was also significantly decreased by TPEN treatment, 0.6-fold (p<0.001) and 0.3-fold (p<0.05), respectively, in the two cell types. *ZnT3* was significantly decreased by TPEN in macrophages (0.3-fold, p<0.001) but not in monocytes while *ZnT7* was significantly decreased in monocytes (0.3-fold, p<0.001) but not in macrophages. TPEN treatment had no significant effect on *ZnT2,4, 8, 9 or 10* mRNA levels (Fig. 2).

### 4. Immunofluorescence localization of ZIP1 and ZIP2 on human macrophages

Two sources of human macrophages were studied, PMA-induced THP1 macrophages and in situ alveolar macrophages from lung sections from non-tumour areas of biopsies obtained from patients undergoing lung tumour resection surgery. Alveolar macrophages were identified by labelling with a mouse monoclonal antibody to CD68.

#### ZIP1

**Figure 3:**
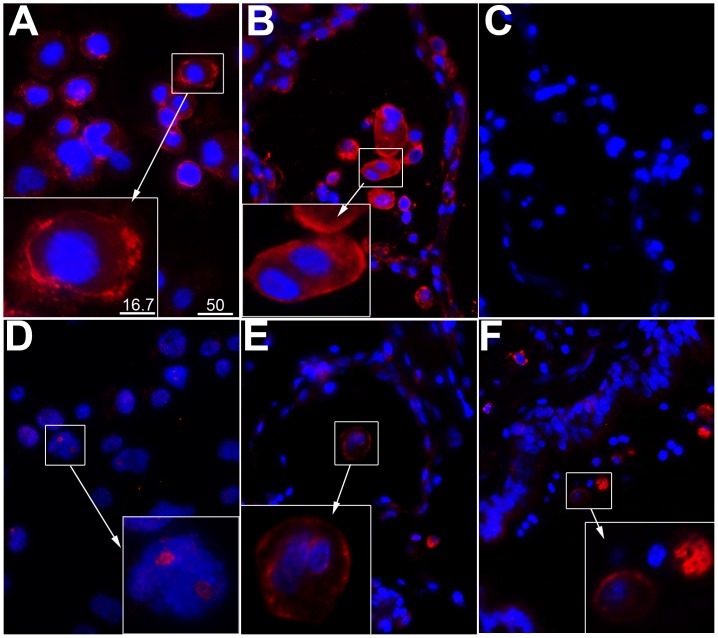
Immunolocalization of ZIP1 and ZIP2 transporters in human macrophages. **A:** Plasma membrane-like localization of ZIP1 (red) in PMA-differentiated THP-1 macrophages. **B**–**C:** Staining of human lung sections (non-tumour area) for ZIP1 (red, B) and negative control (C). **D**: Sparse and predominantly nucleolar localization of ZIP2 in PMA-differentiated THP-1 macrophages. **E**–**F:** Staining of human lung sections (non-tumour area) for ZIP2. Macrophages were identified in separate experiments using CD68 marker (not shown). Panels E and F are representative microphotos of ZIP2 staining (red) of macrophages in two different patient biopsies, showing surface and cytoplasmic fluorescence. Blue = DAPI staining of nuclei. Scale bar represents 50 µm for all microphotos, and 16.7 µm in the insets.

ZIP1 was labelled with a sheep polyclonal antibody. It was strongly expressed in macrophages and localized, predominantly, to the plasma membrane and cytoplasm of both THP1 macrophages (Fig. 3A) and alveolar macrophages (Fig. 3B), consistent with a role as a Zn influx transporter. Negative controls in which the primary antibody was replaced by normal sheep IgG showed no binding of conjugate (Fig. 3C).

#### ZIP2

ZIP2 was weakly expressed on THP1 macrophages and mainly on the nucleoli (Fig. 3D). Localization in the nucleolus was confirmed by Z-section analysis (not shown). On the other hand, there was strong expression of surface and cytoplasmic ZIP2 on alveolar macrophages. This was a consistent finding with several patients’ lung sections, two of which are shown in panels E and F.

### 5. Effect of ZIP1 KO on cytosolic Zn levels and efferocytosis in mouse peritoneal macrophages

These experiments used a ZIP1 null mouse strain. For good yield of macrophages we obtained peritoneal macrophages after ip injection of adjuvant 2 days previously.

#### Cytosolic Zn

**Figure 4:**
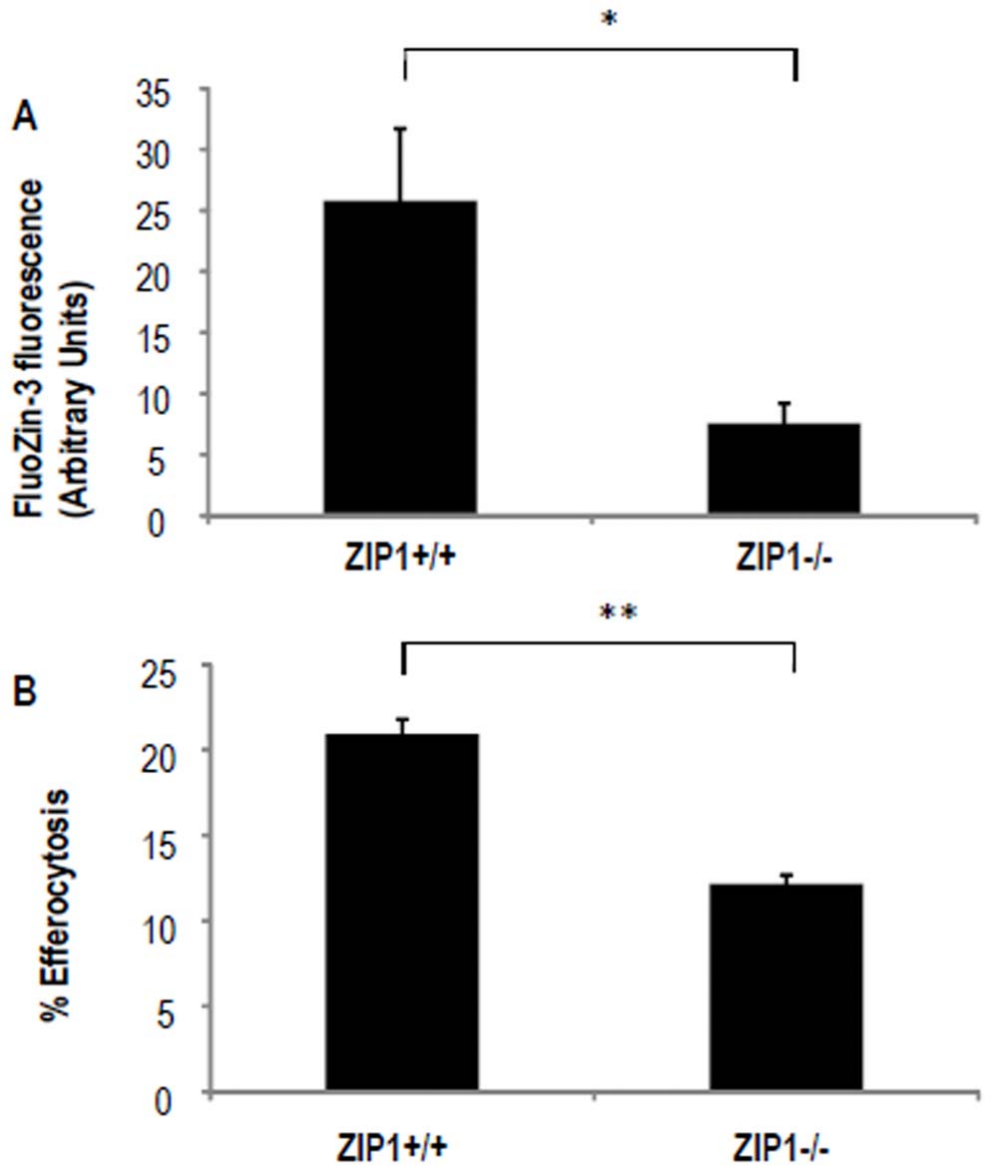
Effect of ZIP1 XO on cytosolic Zn and efferocytosis. Peritoneal macrophages were harvested from WT mice (n = 25) and Zip1 KO mice (n = 14). **A:** Macrophages were stained with FluoZin-3 and the resulting fluorescence was measured by flow cytometry. Positive fluorescence was determined by gating relative to an unstained control. Error bars represent the SEM. **B:** Macrophages were assayed for efferocytosis. Results are expressed as a percentage of macrophages ingesting apoptotic cells. Error bars represent the SEM. * p: <0.05, ** p: <0.005.

Fig. 4A shows that cytosolic Zn (as measured by flow cytometry using the Zn fluorophore FluoZin-3) was significantly (p<0.05) decreased from 25.8% Zn +ve cells (SEM 5.9, n = 25 mice) in the macrophages of ZIP1^+/+^ mice to 7.6% +ve cells (SEM 1.6, n = 14 mice) in macrophages of ZIP1^−/−^ mice. Fluorescence was inhibited by pre-treatment of macrophages for 4 h with 16 µM Zn chelator TPEN (not shown), confirming the Zn dependence of the assay.

#### Efferocytosis

Next we assayed efferocytosis of apoptotic epithelial cells by the peritoneal macrophages as per our published method for AM [5], [6]. With ZIP1^+/+^ macrophages, the efferocytic index was 20.9% (SEM 0.8, data pooled from 2 experiments, n = 14 mice, Fig. 4B). This was significantly (p<0.005) decreased in the ZIP1^−/−^mice to 12.2% (SEM 0.8, data pooled from 2 experiments, n = 12 mice). When the isolated macrophages were depleted of Zn by treatment with 16 µM TPEN for 4 h, the efferocytic index was decreased by 25% in the macrophages from the ZIP1^+/+^ mice but there was no further decrease in efferocytosis in the macrophages from the ZIP1^−/−^ mice (not shown).

## Discussion

Recent studies have highlighted the importance of maintaining sufficient lung Zn levels [16], [17], [27], [37], [38]. In the first section of this manuscript, we report significantly decreased BAL Zn in COPD patients who were current smokers, compared to healthy volunteers, smokers and COPD patients without recent smoking history. It is not clear whether the low lung Zn levels in the actively-smoking COPD patients are a manifestation of a systemic Zn deficiency or whether they indicate a Zn deficiency that is restricted to the lungs. Plasma Zn has been shown to be an unreliable indicator of systemic Zn levels since it is subject to change during the acute phase response in response to inflammation, infection or trauma [39]. In this regard other studies have shown no change, a significant decrease or a significant increase in plasma Zn concentrations in COPD patients compared to healthy controls [40]–[42].

Having shown a Zn deficiency in BAL from COPD patients, we then focussed on the potential role of the zinc deficiency in the defective AM efferocytosis ability that we have previously reported in COPD. Both BAL Zn and AM Zn were positively correlated with the capacity of AM to efferocytose apoptotic bronchial epithelial cells. The link between macrophage efferocytosis ability and Zn was further shown in a murine model: when isolated macrophages were depleted of Zn by treatment with TPEN, the macrophage efferocytic index was decreased by 25%. Importantly this effect was abolished by knocking out of ZIP1, furthermore confirming that Zn was essentially involved. The exact mechanisms by which Zn deficiency impairs efferocytosis are unclear, but current data suggest they are complex. Thus, a pathway of CD44-mediated activation of CR3 which is implicated in phagocytosis and efferocytosis has been shown to be dependent on divalent cations including Zn but not Ca [43]. A further mechanism may involve an impaired differentiation of monocytes to macrophages in Zn deficiency, e.g. due to defective expression of a receptor for GM-CSF [18]. In support of the latter, we have previously shown an increase in undifferentiated macrophages in the lungs of COPD patients who are current smokers compared to never smokers and COPD subjects who had ceased smoking [2]. These smoking related changes may contribute to reducing the overall rate of efferocytosis in the lung, as undifferentiated macrophages have reduced phagocytic ability. It is possible that sustained low BAL Zn levels, as a direct consequence of exposure to cigarette smoke, may lead to a resultant loss of AM Zn by replacement of Zn with Cd in AM [25], although this requires further investigation.

What causes the low lung Zn levels in smokers with COPD is not precisely known although it is possible that cigarette smoke-induced inflammation and lung injury plays a role. In animal models of airway inflammation or acute lung injury, there were abnormalities in airway epithelial or AM Zn transporter expression, including ZIP1, without systemic Zn deficiency [17], [44]. Lung injury and inflammation may therefore lead to a down-regulation in lung Zn transporter expression that lowers Zn concentrations in the lung fluids, airways and AM and results in impaired clearance of dying airway and alveolar epithelial cells.

A gap in the literature is the paucity of information on Zn homeostasis in the macrophage and the mechanisms by which Zn influences critical functions of the macrophage such as efferocytosis. We have therefore begun to characterize those Zn transporters important for monocyte/macrophage Zn homeostasis. Cellular Zn homeostasis involves, in part, the coordinate action of two families of Zn transporters with opposing actions. ZIP transporters increase cytosolic Zn by importing extracellular Zn or by releasing organelle stores of Zn into the cytosol. ZnT transporters export Zn or compartmentalize it into organelles. In particular, there is a need to identify the plasma membrane Zn importers responsible for bringing Zn into the monocytes and macrophages.

One strategy is to look for up-regulation of Zn transporters in response to induced Zn deficiency. Of interest, when the THP-1 monocytes or macrophages were rendered Zn-deficient *in vitro*, by exposing to the membrane-permeable Zn chelator TPEN, there was no change in *ZIP1* expression indicating that this Zn transporter is unlikely to be involved in maintaining cytosolic Zn levels during Zn deficiency. However, mRNA for *ZIP-2* gene increased significantly 28-fold and 3-fold, respectively. This suggests that ZIP-2 is an important Zn transporter for these cells under Zn-deprived conditions. It is interesting that the increase was almost 10-fold greater for monocytes than macrophages. This may indicate that macrophages have more substantial reserves of zinc. Of the three ZIP transporters that decreased at the mRNA level with Zn depletion, ZIP4 is a plasma membrane Zn importer while ZIP5 and ZIP7 are transporters involved in efflux of Zn into the cytosol from ER, Golgi and other organelles. ZIP5 and ZIP7 proteins may, therefore, have a role in maintaining cytosolic Zn levels during Zn deficiency.

None of the ZnT type Zn transporters increased in gene expression in Zn-depleted monocytes or macrophages, while many decreased in expression. Notably, there were large decreases in ZnT1, a transporter that is primarily involved in efflux of Zn out of the cell. Decrease in ZnT1 expression is likely involved in maintaining intracellular Zn when extracellular Zn is limited. There were also decreases in ZnT5-7. Since these are involved in sequestration of Zn into organelles, their decrease might also help to conserve cytosolic Zn.

Joshi and colleagues have previously identified ZIP1 as being important for lung Zn homeostasis and AM phagocytosis of bacteria [17]. We confirmed the presence of ZIP1 protein at the surface, and in the cytoplasm, of THP-1 macrophages and human AM (the latter by immunofluorescence co-localization of ZIP1 and CD68 on macrophages in paraffin-embedded sections of normal lung tissue from patients undergoing lobectomy). Our immunofluorescence studies also showed ZIP2 on the surface and in the cytoplasm of alveolar macrophages. Interestingly, there was only weak, nucleolar-like staining for ZIP2 in THP1 macrophages. Nucleoli are known to be rich in Zn [45]. While presence of ZIP2 in nucleoli requires confirmation by other techniques such as cell fractionation, we believe this is the first report of a candidate nucleolar Zn transporter. The function of nucleolar Zn is unclear but Zn is important for RNA polymerase [46]. Since ZIP1 gene expression is up-regulated in Zn-deprived THP1 cells, the decreased level of ZIP2 protein in THP1 macrophages, as opposed to in situ alveolar macrophages, may be a reflection of higher concentrations of Zn ions in the THP1 culture medium compared to *in vivo* lung fluids. The latter is difficult to measure since the process of obtaining lung fluid lavages introduces a dilution factor.

In this manuscript we also show that both intracellular Zn and efferocytic capacities of macrophages were decreased in ZIP1^−/−^ mice. It was necessary to use peritoneal macrophages for this, as there were limitations on the number of alveolar macrophages that we could isolate. Pregnant ZIP1^−/−^ mice on a low Zn diet were reported to have increased abnormalities in their embryos compared to those of ZIP1^+/+^ mice on the same diet, but no difference was observed when the null and wildtype mice were on Zn replete diets [29]. The authors proposed that ZIP1, and the related transporter ZIP3, modify the effects of Zn deficiency and, therefore, mutations in these genes might impact a wide array of disease processes that are exacerbated by Zn deficiency, including chronic lung inflammatory diseases.

The studies described here begin to address the basic science questions underlying the role of Zn in AM, including the first characterization of the subcellular distribution of Zn and Zn transporters in these cells as well as the role that altered Zn metabolism plays in efferocytosis. We have identified candidate ZIP and ZnT type Zn transporters which may function to maintain cytosolic Zn in Zn deficiency. We have also identified two Zn transporters ZIP1 and ZIP2 which are candidate plasma membrane influx Zn transporters for lung macrophages. ZIP1 was unresponsive to a zinc deficiency signal but was important for normal efferocytosis function. ZIP2 may play a special role in maintaining macrophage Zn in the presence of low extracellular Zn concentrations and this might be important in lung inflammation and injury. Its role, if any, in efferocytosis needs further investigation. Future studies demonstrating that addition of Zn to either monocytes or macrophages increases efferocytosis, and assessing Zn transporter mRNA in human AM will be of importance for better understanding the role of Zn in macrophage dysfunction in COPD. Elucidation of the mechanisms leading to defective efferocytosis in lung injury and disease will provide a foundation for our long term goals of better clinical management of COPD.

## Supporting Information

Checklist S1 **ARRIVE Guidelines Checklist.**
(PDF)
